# Statistical methods for quantitative mass spectrometry proteomic experiments with labeling

**DOI:** 10.1186/1471-2105-13-S16-S7

**Published:** 2012-11-05

**Authors:** Ann L Oberg, Douglas W Mahoney

**Affiliations:** 1 Division of Biomedical Statistics and Informatics, Department of Health Sciences Research, Mayo Clinic, 200 First Street SW, Rochester, MN 55905, USA

**Keywords:** mass spectrometry, experimental design, quality control, normalization, variance structure, labeling, iTRAQ, relative quantification

## Abstract

Mass Spectrometry utilizing labeling allows multiple specimens to be subjected to mass spectrometry simultaneously. As a result, between-experiment variability is reduced. Here we describe use of fundamental concepts of statistical experimental design in the labeling framework in order to minimize variability and avoid biases. We demonstrate how to export data in the format that is most efficient for statistical analysis. We demonstrate how to assess the need for normalization, perform normalization, and check whether it worked. We describe how to build a model explaining the observed values and test for differential protein abundance along with descriptive statistics and measures of reliability of the findings. Concepts are illustrated through the use of three case studies utilizing the iTRAQ 4-plex labeling protocol.

## Background

In this manuscript we focus on statistical methods for quantitative mass spectrometry (MS) based proteomic experiments as they pertain to labeling protocols. Labeling of fragmented proteins (i.e., peptides) allows specimens to be labeled without altering the chemical properties of the peptides, mixed into a single aliquot and then subjected to MS simultaneously. The advantage of the labeling protocol is that specimens can be distinguished in the resulting data by leveraging known properties of the labels. For example, if stable isotopes are used, the known mass shift resulting from extra neutrons together with known naturally occurring distributions of isotopes in the atmosphere are used during the relative quantification step.

Several different labeling protocols have been developed. In iTRAQ labeling, each specimen is labeled with a different amine-specific isobaric tag [1,2]. In ^16^O/^18^O labeling, one specimen is mixed with "light" water containing oxygen in its natural isotopic state (mostly ^16^O) and a second specimen with "heavy" water containing mostly water molecules with the ^18^O isotope that has two extra neutrons. With stable isotope labeling by amino acids in cell culture (SILAC) cells may be grown in "light" or "heavy" medium [3,4] or mice may be fed chow containing carbon in either the natural ("light") ^12^C state or the ^13^C ("heavy") isotopic state [5]. Similarly, with ^15^N labeling, cells may be grown in "light" or "heavy" medium [6,7].

Labeled protocols are appealing for multiple reasons. Mixing multiple specimens for simultaneous MS reduces the total MS machine time needed to perform an experiment. It also eliminates the between MS experiment variation for the specimens assayed together, thus reducing the variation in the study overall. We demonstrate here application of some fundamental experimental design principles, how to assess need for and success of normalization, and how to use statistical models to assess differential protein abundance for a study using data from multiple MS experiments.

There are three common objectives in high dimensional studies that produce data on a large number of endpoints such as global proteomics studies [8]. *Class comparison *involves comparing abundance levels between predefined groups. An example of this is comparing protein abundance levels between cancerous and benign tumors in order to gain biological insight into the mechanism of cancer. *Class prediction *involves development of a prediction rule consisting of a panel of biomarkers that are useful for classifying a new subject into pre-determined classes such as cancer or benign. Building on the cancer example, this process would combine multiple proteins present at differing abundance levels between cancer and benign tumors in this case, into a prediction rule that could be applied to a new subject with a tumor to determine whether the tumor was benign or cancerous. *Class discovery *involves use of abundance profiles to uncover yet unknown biological subtypes of disease. For example, in a proteomics study of high-grade serous ovarian cancers, the protein abundance data would be used to determine whether subtypes of serous cancer may exist that are currently unknown. The methods of this manuscript are focused on the class comparison objective.

In general we will use *specimen *to refer to the sample material labeled, *tag *to refer to the label applied to the specimen, *experiment *to refer to the set of specimens mixed and subjected to MS simultaneously, and *study *to refer to the collection of MS experiments used to test a particular hypothesis. We assume that protein and peptide identification has already been performed, and that a list of peptides, the associated proteins and abundance levels are available for analysis. Case studies will be used to demonstrate the principles discussed. The beginning portions of the "Assessing the need for and success of normalization" and " Estimation of model parameters and calculating significance" sections will likely be more accessible to statisticians than to non-statisticians; the case studies in those sections provide tangible examples of the concepts being discussed which will likely be more tangible to clinicians and practitioners of mass spectrometry.

## Methods

### Overview

**Figure 1:**
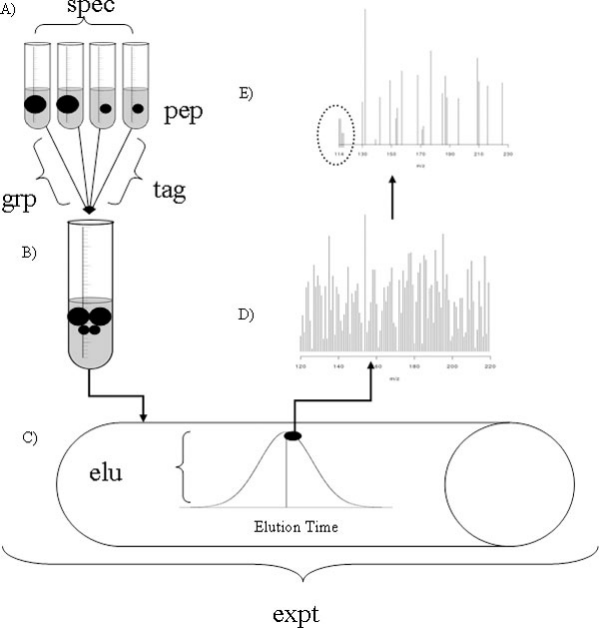
**Cartoon depiction of the 4-plex iTRAQ labeling protocol for one MS experiment**. A) Four specimens are each labeled with one of the four tags. The black dots indicate a given peptide that is present in different relative abundance according to size. B) The four specimens are then mixed into a single aliquot for simultaneous MS analysis. The resulting data constitute an MS experiment. C) Each peptide will take some amount of time to elute off of the LC column and so may be observed multiple times. D) In the first MS the top species according to abundance are chosen for a second MS. It is common for the top 3 or 5 to be chosen. E) During the second MS the iTRAQ tags are broken off and used for relative quantification (left in the dotted circle). It is these data that are used in downstream statistical analyses. The remaining peptides are fragmented further for identification purposes (right).

We utilize three 4-plex iTRAQ data sets as case studies throughout the manuscript. The iTRAQ 4-plex labeling protocol involves adding one of four amine specific isobaric labels which do not alter mass (e.g., 114, 115, 116, or 117) to each of four specimens for simultaneous mass analysis via tandem mass spectrometry. The four mixed specimens are not discernible in the first MS where the most abundant species in the chamber are chosen for relative quantification (see Figure 1). During the second MS, the isobaric tags are broken off and quantification is performed based on the relative abundance of these tags. An 8-plex iTRAQ protocol is also available. See the "Discussion" section for an example of how other labeling protocols may differ.

Here we provide a very brief explanation of each case study. Highly abundant proteins were removed in the GCM and prostate cancer studies, proteins were digested for all three studies, and fractionation was performed via strong cation exchange (SCX) in all three studies.

### Giant cell myocarditis (GCM)

The study focused on three histologic subtypes of acute cardiomyopathy: 1) idiopathic dilated cardiomyopathy (DCM), 2) giant cell myocarditis (GCM) and 3) lymphocytic myocarditis (LM). These three subtypes present with similar clinical symptoms. However, GCM is much more lethal and requires a very different treatment strategy. Immediate objectives included comparing protein abundance profiles between these groups and long-term objectives included finding a protein present in blood useful as a diagnostic tool.

Six subjects of each subtype were included in the study. Though less than ideal (rationale will be discussed more in later sections), a pool of six normal healthy controls was used as a reference (N). Specimens were mass analyzed via capillary reverse-phase LC/MS/MS on a QSTAR quadripole time of flight mass spectrometer. Protein identification was performed via ProQuant. A total of six MS experiments were performed. Full experimental details are available elsewhere [9,10].

### Prostate cancer

This study used serum from prostate cancer patients to understand changes from pre- to post-androgen deprivation therapy (ADT) (n = 15 paired specimens) and to understand the differences between subjects experiencing ADT failure within a short (n = 10) versus long (n = 10) time-frame. Mass analysis was performed with an LTQ-Orbitrap Velos mass spectrometer. Final analyses are still being performed, so group membership is blinded for the current manuscript. A total of 13 MS experiments were performed. Two of the experiments were run a second time as indicated by an 'R' suffix (1R and 13R).

### Yeast spike-in

A spike-in study was performed using yeast lysate to represent a complex background with the goal of understanding variance structure, systematic experimental biases and ability to detect fold changes of various magnitudes. Sixteen proteins with masses ranging from approximately 11 to 98 kDa were combined into two spike-in mixes; each protein was present in one mix at a "low" concentration and in the other mix at a "high" concentration. Each mix was then spiked into the yeast background at relative concentrations (fold changes) ranging from 1.0, 1.1, 1.2, 2.0, and 5.0. For each mix, two combinations of fold changes were performed: 1.0 : 1.5 : 1.0 : 5 and 1.1 : 1.0 : 2.0 : 1.2. Each of these was mass analyzed in duplicate for a total of eight MS experiments (2 mixes * 2 fold change layouts * 2 replicates). The yeast background was present at equal abundance (1.0 : 1.0 : 1.0 : 1.0) in all experiments. Mass analysis was performed on an LTQ Orbitrap. Full experimental details are available elsewhere [11]. These data are publicly available from http://ProteomeCommons.org Tranche using the following hash search: YW9yck8PKhd5vyKwUt0AIfVVllgXP9RoM0qTZDWQ05aNtae8uIHN/ 1Ird7APnNweSfqjVb9n5fT+oEyfqnOKZdRz3AUAAAAAAAAB8Q==.

## Statistical experimental design

### Overview

The primary goals of statistical experimental design are to maximize information gain while minimizing resource expenditure and avoiding bias. Thoroughly considering the key aspects of replication, randomization and blocking prior to running an experiment ensures that enough of the necessary data is collected in a manner that ensures proper conclusions. In this "Statistical experimental design" section we first briefly describe the issues of bias and variability followed by discussion of the fundamental experimental design strategies to combat these issues.

### Bias

Bias is any trend in collection, analysis, interpretation, publication or review of data that can lead to conclusions that are systematically different from the truth. A confounded factor is one that is associated with both a real causal factor and the outcome of interest [12]. Bias and confounding may enter a study if samples in the comparative classes differ systematically on factors that affect the outcome. Dr. Ransohoff defines bias, describes ways to avoid it, and how to assess it and address it in various types of studies [13].

### Variability

There are several levels of variability including *technical*, *biological *and *institutional*. *Technical *variability deals with reproducibility of an assay. Sample extraction, label, dye, technician, machine, reagent batch are all potential sources of assay variation and could alter the result produced in multiple assays of the same specimen. *Biological *variation is due to the difference between human subjects in a human study, mice in a mouse study, or Petri dishes/beakers of cell line in a cell line study. *Institutional *variation is due to differences between institutions and can be due to differences in patient populations seen, e.g. differences in disease severity or ethnicity, and differences in sample procurement protocols and implementation (even if identical on paper). These levels of variability all play a role in distinguishing signal from noise as well as in the generalizability of study conclusions. In general, technical variability is smaller than biological variability, which in turn is smaller than institutional variability. Generally, biological variability is the focus of most studies.

### Replication

One of the main threats to validity and generalizability of experiments where a large number of endpoints are measured on a small set of subjects is chance [13]. Replication is the tool that increases the precision of study conclusions and reduces the possibility that they are due to chance. There are several levels of replication that parallel the levels of variability. *Technical replication *involves repeated assays on the same biological replicate. This could involve one extraction of sample material undergoing sample preparation procedures as a unit but subjected to assay multiple times. It could also involve more than one extraction of sample material with each extraction then undergoing the sample preparation process on its own. *Biological replication *involves studying multiple members of the population being studied. For example, in a human study, each person in the study constitutes one biological replicate. If each human provides, say both cancer tumor tissue and normal tissue, then the pair of cancer-normal specimens constitutes one biological replicate. In an animal study, each animal constitutes one biological replicate. In a cell line study, each dish of cells grown up and subjected to treatment on its own constitutes a biological replicate. *Institutional replication *involves a study being performed at multiple institutions.

The optimal replication strategy depends on the goal of a study. A study with the goal of understanding and estimating sources of assay variability requires various types and levels of technical replication on a small number of biological replicates. Class comparison and class prediction studies have the goal of better understanding distinct classes of subjects. Study results are generally inferred back to population classes of subjects, making it ideal to maximize the precision of statements about those populations. Technical replication increases the information and precision about a specific subject while biological replication increases the information and precision about a population. Thus, the greatest information gain and increase in precision for inferences to the study population comes from allocating available resources to more biological replicates rather than technical replicates. The mathematics supporting this are demonstrated elsewhere [14].

In practice, it is wise to include technical replicates on a few of the biological replicates in high dimensional experiments, especially if the assay platform or protocol is new to the laboratory, for use in evaluating and reporting on reproducibility and quality. Institutional replication is often utilized in studies with validation as the goal.

### Blocking

Statistical blocking is a tool that helps to guard against known potential biases and to minimize variance in a study. Blocking is sometimes referred to as matching in the context of sample selection, where for example, subjects are matched on gender or paired specimens are taken from the same subject. In the context of spectral acquisition, blocking is sometimes referred to as multiplexing. Specimens assayed within a block are more similar than specimens assayed between (in different) blocks. Use of this strategy in allocating specimens to MS experiments and tags is called a Randomized Block Design (RBD). MS experiment is a natural blocking factor in labeled work-flows and should be used as such. Labels or tags, day of MS assay, laboratory technicians, reagent batches, MS machines or LC columns are other examples of natural blocking factors. To protect against bias, avoid confounding and minimize variance about the question of interest, some specimens from each study group should be allocated to be assayed together within a block. This is the basis of the RBD and is demonstrated in the case study examples towards the end of this section on "Statistical experimental design". A labeled MS study with only one MS experiment will result in study groups being confounded with labels and very small sample sizes. It is good practice to utilize multiple MS experiments in order to avoid confounding of study groups and tag effects and reasonable sample sizes.

### Randomization

Randomization is a tool that protects a study from both known and unknown biases. This tool is utilized during both subject selection and during the allocation of specimens to sample processing order. Randomized selection of subjects generally ensures that potential biases which may influence the outcome are approximately balanced across the study groups and is discussed in greater detail elsewhere [15,16].

Randomized allocation of study specimens over assay run order generally ensures group membership is approximately balanced over run order, thereby eliminating the potential confounding of study group and run order. In a labeled workflow using MS experiment as a blocking factor, this allocation takes place in two steps. Consider the 4-plex iTRAQ workflow and a study with four groups of interest such as the GCM study. Thus, the number of groups is equal to the number of tags within each MS experiment block. The first step is to allocate one specimen from each study group to each block. To do this, a random number is generated for each biological replicate via a random number generator, such as the RAND function in excel. These numbers are then ranked within study group to determine which specimen is allocated to MS experiment 1, 2, etc. The second step is to allocate specimens to labels within a block. This can be done using the same random number, or a second random number could be generated, with the rank order of these random numbers determining the tag allocation.

Though a consistent tag bias affecting all proteins has not been demonstrated in iTRAQ data, there are likely protein-specific tag biases. Thus, it is wise to ensure tag and study group are not confounded. Check the randomization to be sure groups are approximately balanced over tag so that group and tag are not confounded. Alternatively, both MS experiment and tag can be used as blocking factors. This is especially wise in studies with very small sample sizes.

#### Case study: GCM data

Both MS experiment and labeling tag were used as blocking factors in this study. First, one specimen from each of the four study groups was allocated to an MS experiment. Second, within each MS experiment, the four specimens were randomly assigned to a tag so that the study groups were approximately balanced over tags. Both steps were accomplished using a random number generator. See Table 1 for the resulting allocation. Though the normal pool was included as a reference, it was randomly assigned to tag within a block in order to avoid confounding of tag and study group. As a result of the blocked randomization, any potential effects or biases due to tag can be distinguished from study group using a statistical model for differential abundance. This will be discussed in more detail in the "Differential abundance" section.

**Table 1:** Statistical experimental design of the GCM study demonstrating allocation of specimens to MS experiments and labeling tags.

Experiment	Tag			
	
	114	115	116	117
1	GCM1	DCM1	LM1	Normal Pool1
2	DCM2	Normal Pool2	GCM2	LM2
3	Normal Pool3	LM3	GCM3	DCM3
4	LM4	GCM4	Normal Pool4	DCM4
5	DCM5	GCM5	Normal Pool5	LM5
6	Normal Pool6	DCM6	LM6	GCM6

(Adapted with permission from [9]. Copyright 2008 American Chemical Society.) The abbreviations GCM, DCM, LM and N (normal control pool) denote the four groups under investigation as described in Section 2.2. The numbers denote biological replicates for GCM, DCM and LM, and technical replicate number for N. For example, GCM1 is the first sample in the GCM group. Experiment number also corresponds to run order.

The rationale for using a pool as a reference in a labeled design is based on the fact that the abundance measures are relative and the pool can be used as a normalizing factor of sorts to adjust for technical variation. With this strategy, abundance values are divided by the pool abundance values to create a "normalized" ratio. First, this strategy assumes the normalization factor is identical for each specimen within the MS experiment. However, normalization factors generally differ for each specimen due to slight but non-ignorable differences in sample handling from the time of extraction from the subject to mass analysis. Second, the resulting ratios are generally ill behaved and difficult to deal with in statistical analyses. This will be discussed further in the "Data quality and normalization" and "Differential abundance" sections. Third, this induces a correlation between observations, violating the independence assumption of statistical tests. Model-based methods for normalization are described in the "Data quality and normalization" section. Fourth, it is not possible to correctly perform statistical differential abundance between the six normal specimens in the pool and other study groups since biological variability cannot be estimated for the normal specimens. Statistical designs and the associated analysis methods were developed specifically to deal with relative measurements in the early 1900's[17,18], obviating the need for a reference sample in each MS experiment.

#### Case study: prostate data

Two comparisons were of interest in the prostate cancer study. The first comparison was between pre- and post-ADT treatment protein profiles in paired specimens from each of 15 patients in order to understand proteins indicating early response to ADT. The second comparison was between ten subjects who failed ADT within 12 months (short) and ten subjects who failed after 30 months (long). In addition, for proteins found to be significantly differentially a in the pre- to post-ADT comparison, the investigator wished to assess behavior of those proteins in the short and long cohorts. Thus, it was important to keep paired pre and post specimens within the same MS experiment in order to minimize variability in that comparison. Second, it was important to allocate at least one short and one long specimen to the same MS experiment in order to minimize variability in that comparison. Third, it was important to observe most of the proteins in both sets of subjects. Thus, given the data-dependent acquisition process of global MS studies, it was important to include both pre/post specimens together with short-term/long-term in the same MS experiments.

The randomization plan accounted for these goals. Thirteen MS experiments were required to assay the 50 specimens and two technical replicates. First, one short-term and one long-term subject were randomly assigned to 10 of the 13 MS experiments, allocating all 20 of these specimens. Second, a pair of pre/post specimens was randomly assigned to those same 10 MS experiments, allocating 10 of the 20 pairs of specimens. Third, the remaining five pairs of specimens were randomly assigned to the remaining three MS experiments. Fourth, the four specimens assigned to each MS experiment were randomly assigned to tag, ensuring balance of the groups over tag. See Table 2 for the resulting allocation.

**Table 2:** Statistical experimental design of the prostate cancer study.

Experiment	Tag			
	
	114	115	116	117
1, 1R	Pre	Late	Early	Post
2	Post	Early	Late	Pre
3	Early	Post	Pre	Late
4	Post	Early	Late	Pre
5	Late	Pre	Post	Early
6	Late	Early	Post	Pre
7	Pre	Post	Early	Late
8	Pre	Pre	Post	Post
9	Early	Late	Pre	Post
10	Post	Post	Pre	Pre
11	Post	Pre	Late	Early
12	Early	Pre	Late	Post
13, 13R	Post	Late	Pre	Early

Statistical experimental design of the prostate cancer study demonstrating allocation of specimens to MS experiments (where an 'R' suffix indicates that experiment was re-run) and labeling tags. The abbreviations Pre, Post, Early and Late denote the four groups under investigation as described in Section 2.3, pre-ADT, post-ADT, ADT failure within a short time-frame, ADT failure within a long timeframe. The numbers denote biological replicates for each group. For example, Pre1 is the first sample in the pre-ADT group.

## Data quality and normalization

### Obtaining the data

Vendor software generally creates data reports in which abundance data has been divided by the abundance in one specimen or tag that is designated as the reference. This reference specimen may be a control or a pool, or represent one of the study groups of interest. However, ratios are generally ill behaved, and it is preferable to work with the individual abundance values in statistical analyses [14,19]. For example, when abundance values in the control are very small, the resulting ratios get incredibly large very quickly due to very small numbers in the denominator. In addition, such ratios are not immune to pipetting errors or differences in specimen processing.

Thus, it is preferable to work with data that have not been put into a ratio format. That is, we want the peptide level abundance values for each labeled specimen for use in statistical analyses. It is not always obvious how to obtain this data. In the ProteinPilot software with which we are familiar, individual reporter ion area under the curve values are contained in the Peptide Summary exports. These reports are generated by first opening the results file (*.group) in ProteinPilot and then clicking on Peptide Summary export on the left side of the page. The user is then prompted for a location to save the resulting .txt file. The desired data are near the last columns in the spreadsheet and are given variable names such as Area114, ..., Area117.

An a priori list of proteins does not exist for global MS studies. Rather, the goal is to catalogue as many proteins as possible in a specimen and obtain quantification information for them. A "divide-and-conquer" strategy is employed since MS instruments have a dynamic range of around 4-5 orders of magnitude while the human proteome spans over 12 [20]. A specimen undergoes many steps in this process including digestion to break proteins into peptides and fractionation to separate the specimen into less complex sub-samples via some chemical property such as charge state (saltiness) and/or hydrophobicity (ability to mix with water) [21,22]. As material is introduced into the mass spectrometer, generally only the most abundant species are selected for MS, e.g., the top three or five. Thus, the data acquisition is abundance-dependent. As a result, iTRAQ studies using multiple MS experiments typically have many proteins/peptides that are not observed in all MS experiments. Due to the dynamic range of the proteome, whether human or other species, approximately half of the species in a specimen are present at the level of detection. So even in technical replicate MS experiments there can be a large number of proteins which are not observed in both experiments.

The tandem MS is utilized in iTRAQ to choose a species in the first MS and then perform identification and quantification in the second MS, generally resulting in an observed abundance value for all for specimens within an experiment. Thus, there is generally not missing data for a given peptide within an MS experiment. This has implications for the normalization strategy. See Table 3 for an example of a typical data matrix.

**Table 3:** Snapshot of an iTRAQ data table.

MSMS Spectrum ID	Protein Accession	Peptide Sequence	114	115	116	117	Experiment Number
S4_F11.1140.1140.2	GPP1_YEAST	(F)EDAPAGIAAGK(A)	2813.568536	1595.741524	2475.724121	2458.306255	4
S1_F16.2850.2850.3	GPP1_YEAST	(K)GRNGLGFPINEQDPSK(S)	316.4418979	466.2738416	630.4750319	444.921289	1
S3_F16.2618.2618.3	GPP1_YEAST	(K)GRNGLGFPINEQDPSK(S)	869.2210037	544.1843783	1617.949095	665.3067241	3
S3_F16.2623.2623.3	GPP1_YEAST	(K)GRNGLGFPINEQDPSK(S)	1163.021548	925.1491063	1347.204837	1032.958433	3
S1_F13.1643.1643.2	GPP1_YEAST	(K)DDLLK(-)	10607.97083	8544.75492	10953.83841	9005.777375	1
S1_F13.1513.1513.2	GPP1_YEAST	(K)DDLLK(-)	1748.258583	2893.388823	1861.30691	2715.653088	1
S1_F13.1507.1507.2	GPP1_YEAST	(K)DDLLK(-)	606.7841803	919.8748238	1144.338397	1025.119065	1
S1_F13.1643.1643.2	GPP1_YEAST	(K)DDLLK(-)	10607.97083	8544.75492	10953.83841	9005.777375	1
S2_F13.1291.1291.2	GPP1_YEAST	(K)DDLLK(-)	2618.558021	1367.979923	2947.928581	2321.749983	2
S2_F13.1291.1291.2	GPP1_YEAST	(K)DDLLK(-)	2618.558021	1367.979923	2947.928581	2321.749983	2
S3_F13.1582.1582.2	GPP1_YEAST	(K)DDLLK(-)	1849.138156	2882.532646	3456.336093	3333.133633	3
S3_F14.1374.1374.2	GPP1_YEAST	(K)DDLLK(-)	88.57809719	39.54738544	113.4348917	128.6087568	3
S3_F13.1360.1360.2	GPP1_YEAST	(K)DDLLK(-)	5897.197655	8115.16893	5413.842313	6349.146183	3
S3_F13.1357.1357.2	GPP1_YEAST	(K)DDLLK(-)	3232.418762	6524.148517	5246.904457	5391.07817	3
S3_F13.1582.1582.2	GPP1_YEAST	(K)DDLLK(-)	1849.138156	2882.532646	3456.336093	3333.133633	3
S3_F14.1374.1374.2	GPP1_YEAST	(K)DDLLK(-)	88.57809719	39.54738544	113.4348917	128.6087568	3
S3_F13.1360.1360.2	GPP1_YEAST	(K)DDLLK(-)	5897.197655	8115.16893	5413.842313	6349.146183	3
S3_F13.1357.1357.2	GPP1_YEAST	(K)DDLLK(-)	3232.418762	6524.148517	5246.904457	5391.07817	3
S4_F13.1395.1395.2	GPP1_YEAST	(K)DDLLK(-)	2404.371623	2571.938103	4057.845902	3907.827732	4
S4_F13.1399.1399.2	GPP1_YEAST	(K)DDLLK(-)	3195.952185	3638.020997	6349.053364	6973.840279	4
S4_F13.1395.1395.2	GPP1_YEAST	(K)DDLLK(-)	2404.371623	2571.938103	4057.845902	3907.827732	4
S4_F13.1399.1399.2	GPP1_YEAST	(K)DDLLK(-)	3195.952185	3638.020997	6349.053364	6973.840279	4

This table shows a snap shot of an iTRAQ data table from the yeast data.

### Assessing the need for and success of normalization

Observed abundance values produced by global mass spectrometry machines are relative rather than absolute. In addition, experimental effects between MS runs have been demonstrated in several proteomic work-flows [23]. Even in labeled work-flows which reduce between MS experiment variability, abundance values are subject to other experimental factors such as sample handling from the time the specimen was extracted from the subject, pipetting errors or other potential sources of bias [24]. Thus, data must generally be normalized prior to performing comparisons between groups of interest.

Normalization via standard curves is problematic in these experiments that catalogue and quantify hundreds to thousands of proteins in a single assay. However, normalization methods have been developed utilizing the entire data distributions. These make some specific assumptions about the data. Most algorithms assume: 1) only a small portion of the proteins are differentially abundant between groups of interest, 2) the fold change distribution of differentially abundant proteins is symmetric about 1.0, 3) data must be available on a sufficient number of proteins with abundance levels distributed throughout the dynamic range to estimate global biases without over-fitting [25]. For example, quantile [26,27] and cyclic loess normalization [28-30] are examples of normalization algorithms developed for one- and two-color gene expression arrays that make these assumptions. The iterative ANOVA model [9] described in the "Data quality and normalization" section is an example of such a normalization algorithm which can be applied to both labeled and label-free proteomics abundance data.

There are several visualization tools which are useful for assessing data quality, the need for normalization and the success of normalization. These include peptide or protein coverage plots, box-and-whisker plots (box plots for briefness), and minus versus average (MVA or MA) plots. We define these and provide some examples of each in subsequent paragraphs.

Peptide and protein coverage plots are useful for understanding the magnitude of missing data in a data set, and how many peptides/proteins were detected in multiple MS experiments. They can highlight systematic effects present in the data for further investigation. The axes indicate MS experiment number versus some rank order of the peptide or protein ID. The sort order of the peptides can be by average abundance, by number of experiments it was observed in, or other. A line is placed on the plot if the peptide was detected in that experiment, white space if it was not detected. A peptide that was detected in all MS experiments in a study would show as a solid line across the entire plot.

Box plots provide a visual summary of a distribution. The bottom, mid and top lines of the box represent the 25^th^, 50^th ^(median) and 75^th ^percentiles of the distribution. A "whisker" extends above the box to 1.5 times the inter quartile range (i.e., the distance from the 75^th ^percentile to the 25^th ^percentile) or to the maximum value in the distribution, whichever is smallest. Similarly, a whisker extends below the box the same distance or to the minimum value, whichever is largest. If points exist beyond these whiskers, they are represented by dots. There is one box-and-whisker for each specimen in the study. Global biases which affect all peptides are indicated by shifts up or down in the box-and-whiskers. Usually such a shift is not expected due to the disease, i.e., a global increase or decrease in protein concentration in the biological subject is not expected. The sort order of the boxes can be chosen strategically. For example, sorting by MS experiment first and then by tag would help the eye identify global experiment effects whereas sorting by tag first and then experiment would help the eye identify global tag effects. Changes in dynamic range are evident from compression or expansion of the box and whiskers. If normalization has effectively removed global biases, the box plots of post-normalization data should demonstrate similar per-specimen box and whiskers. They typically demonstrate less variability than in the pre-normalization plots as well, as evidenced by reduced height of the box and whiskers.

Minus versus average (MVA) plots are useful for assessing whether bias is a function of mean abundance. Nonlinear bias of this type is common in gene expression data from both single and multi-channel arrays [30,31]. Traditional MVA plots demonstrate agreement in the global distributions (or lack thereof) for two specimens, have the average of the two on the horizontal (x) axis and the difference between the two on the vertical (y) axis, and a point for each peptide or protein that is observed in both specimens. If two replicates yielded identical results, all points would lie on the y = 0 horizontal line (indicated on the plots for reference). Residual MVA plots are advantageous because they allow one plot for every specimen (rather than all pair-wise combinations) and demonstrate visually how a specimen is similar to or different from the average of the others. Here, the horizontal axis is the average over all specimens instead of the average of two specimens and the vertical axis is the difference between that specimen and the average over all specimens.

#### Case study: yeast data

**Figure 2:**
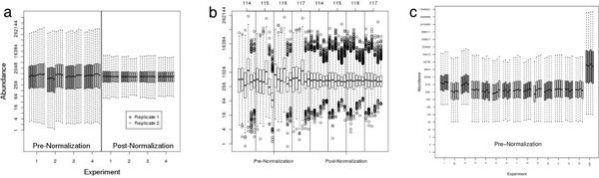
**Box plots of global protein abundance distribution**. Box plots for A) the yeast spike in study, B) the GCM study and C) the prostate cancer study. The log2 scale raw mass spectrometric signal abundance (labeled on the raw scale) is plotted as a function of MS experiment number. Sort order in A is first by MS experiment and then by tag, and boxes are grouped in sets of 8 where the first four (dark grey) are the first technical replicate MS experiment and the second four (light grey) are the second technical replicate MS experiment for a given spike-in combination. Sort order in B was chosen to be first by tag and then by MS experiment in order to help assess whether a systematic tag bias was present. Sort order in C is first by MS experiment and then by tag. (Panel B is reproduced with permission from [9]. Copyright 2008 American Chemical Society.)

Pre-normalization box plots of peptide abundance values from the yeast study demonstrate that, even in a well-controlled experiment where all but 16 proteins are present at 1.0 : 1.0 : 1.0 : 1.0 ratios, between MS experiment and tag effects exist (see Figure 2a, left panel). Post-normalization box plots (see Figure 2a, right panel) demonstrate that the global distributions have similar percentiles and the variability has been reduced, both indicators of successful normalization.

**Figure 3:**
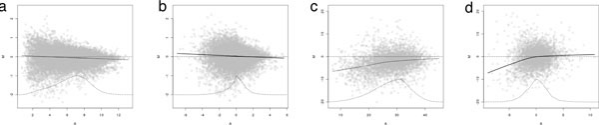
**MVA plots**. Pre- (panel A) and post-normalization (panel B) within-experiment MVA plots. Pre- (panel C) and post-normalization (panel D) between-experiment MVA plots. The vertical axis is difference between the intensities in two specimens on the log2 scale and the horizontal axis is the average of the two intensities on the log2 scale (note the different in axes labels between the top and bottom plots); there is one point for each peptide observed in both specimens. A locally weighted moving average smoother is indicated to demonstrate the average bias curve as a function of average abundance. A smoothed histogram is included at the bottom of the plots to demonstrate the number of data points represented directly above that area in the plots.

MVA plots in the yeast study demonstrate a small amount of global shift in abundance (see Figure 3), more between MS experiments than within as would be expected. The fact that the smoother is shifted away from the y = 0 line indicates global bias. The curvature in the smoother indicates the bias may be abundance-dependent. If normalization has been effective at removing global biases, the smoothers on post-normalization MVA plots should overlay the y = 0 line. This is nearly true in these data. Some nonlinearity remains post-normalization. However, these are in a region where there are very few data points as demonstrated by the smoothed histogram at the bottom of the plot. Completely removing this bias would be viewed as over-fitting the data. Most experimental biases we have seen in iTRAQ data have been mostly linear in nature, but this should be evaluated on a per-study basis.

**Figure 4:**
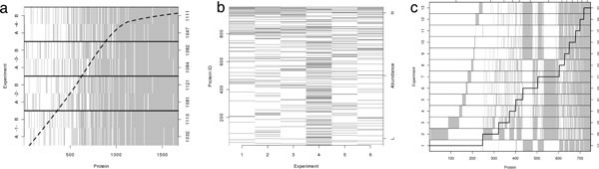
**Protein coverage plots**. A) Protein coverage plot for the yeast study. The left vertical axis indicates MS experiment while the right vertical axis indicates the number of proteins observed in each MS experiment. The horizontal axis indicates the protein rank when sorted by average abundance. The dashed smoother indicates average number of MS experiments in which proteins in that region were detected. B) Protein coverage plot for the GCM study (Reprinted with permission from [9]. Copyright 2008 American Chemical Society.) The left vertical axis indicates rank average protein abundance while the right vertical axis indicates sort order of abundance. The bottom axis indicates MS experiment number. C) Protein coverage plot for the prostate cancer study. The left vertical axis indicates MS experiment while the right vertical axis indicates number of proteins observed in each experiment (experiments 1R and 13R are not included here). The bottom horizontal axis indicates rank of protein ID, where proteins are sorted by the number of experiments they were detected in. The stair-step line helps the eye to delineate which proteins were observed in 1 MS experiment (first step on the far left) up to all experiments (top step on the far right) while the top horizontal axis indicates the number of proteins represented by each step.

The abundance-dependent data acquisition process is evident in a protein coverage plot for the yeast data through the gradation of shading; there are fewer proteins present on the left at low abundance levels than on the right at high abundance levels (see Figure 4a). It is also evident that a larger portion (relative to the other case studies) of proteins were observed in most of the MS experiments in this well controlled spike-in study.

#### Case study: GCM data

The coverage plot from the GCM study demonstrates that many more peptides were detected in experiment 4 than the other experiments (see Figure 4b). In discussing the results with the researchers, we learned that experiments 1-3 had been performed within a short time-frame, experiment 4 was performed approximately two months later followed by another gap in time before experiments 5 and 6 were performed. Pre- and post-normalization box plots (see Figure 2b) demonstrate linear biases have been removed and variability reduced through normalization.

#### Case study: prostate data

Protein coverage plots from the prostate study (See Figure 4c) indicate a systematic difference between experiments (1, 9-13) and (2-8) as demonstrated by the blocks of proteins present in all of one set of experiments or the other. Upon discussion with laboratory personnel including the mass spectrometry expert and the bioinformatics expert, we determined that a change in the protein identification labels had occurred in between the eighth and ninth MS experiments (experiment 1 was actually run between numbers 8 and 9). This change resulted in protein names represented two different ways for a subset of proteins. Once the naming conventions were applied similarly across all experiments, these "blocks" of proteins were no longer evident.

Box plots from this study demonstrate that the distributions for experiments 1, 2 and 13R (recall the 'R' suffix indicates a repeated MS experiment) were shifted up relative to the other experiments in the box plot (see Figure 2c). In talking with the mass spectrometry expert, there was no known explanation for the shifts in experiment 1 and 2, and review of the spectra deemed the data to be of good quality. Through the discussion we determined that a machine setting had been changed prior to experiment 13R resulting in a nearly 10 fold increase in abundance and far fewer proteins observed compared to other experiments, thus the data was rendered not useable. Experiment 1R was done due to questionable quality of Experiment 1. Thus, the MS experiments used statistical analysis were 1R, 2-13.

### Building the normalization model

Vendor software generally applies a normalization factor within an MS experiment which results in equal median fold changes between the chosen reference specimen and the remaining specimens. This is not adequate with the abundance-dependent data acquisition process [32]. Here, we describe how to build a model for normalization.

We use the observed data, *y*, to indicate the true abundance. However, the observed values are influenced by multiple factors. There are both known biological and experimental factors as well as unknown factors which can be put into a statistical model. Biological factors include study group, subject or specimen, protein and peptide. Experimental factors include MS experiment, tag and elution time (see Figure 1). On the raw scale effects are generally considered to be multiplicative. Thus, the model can be written as

$$y_{ijkpm}=expt_{i}\times tag_{j}\times spec_{ij}\times grp_{k}\times prot_{p}\times pep_{kpm}\times err_{ijkpm}$$

where, *y_ijkpm _*is the observed abundance value, *expt_i _*indicates the *i*th MS experiment, *tag_j _*indicates the *j*th labeling tag, *spec_ij _*indicates the *ij*th specimen (which is also the *expt_i _*× *tag_j _*interaction), *grp_k _*indicates the *kth *study group, *prot_p _*indicates the *p*th protein observed in the *i*th MS experiment, *pep_kpm _*indicates the *m*th peptide observed for the *p*th protein in the *i*th experiment and *err_rijkpm _*indicates random, unspecified error. Note that subscripts may be helpful for some readers. For others, it is important simply to understand the conceptual framework of representing known effects in the model to explain sources of variability in the data. A complete discussion of model terms and the rationale for each can be found elsewhere [33].

The most common and simplest statistical models are based upon additive rather than multiplicative effects. Since it is generally easier to transform data to obtain the proper scale for the mean and then worry about how to model the variance in that framework, the data are generally transformed to the log scale. Log2 is commonly used since it is easy to interpret in your head with differences of 1, 2, 3, etc. corresponding to fold changes of 2, 4, 8, etc., respectively (powers of 2). On the additive scale then, this model can be written as

$${\text{log}}_{2}\left(y_{ijkpm}\right)=expt_{i}+tag_{j}+spec_{ij}+grp_{k}+prot_{p}+pep_{kpm}+\varepsilon_{ijkpm}$$

where the *ε_ijkpm _*are assumed to identically and independently distributed according to a Gaussian distribution. This is the basis of the analysis of variance (ANOVA) model, explaining the sources of variation. Experimental factors are not of interest specifically, but should be accounted for in order to minimize variability and ensure accurate conclusions. Conceptually, including terms such as MS experiment in the statistical model performs group comparisons within an experiment, and then averages these comparisons over all experiments in the study to achieve a unified result based on all available data. It is this concept that allows multiple MS experiments to be combined for unified analysis.

The experimental effects serve as the normalization portion of the model, and the biological effects serve to test the hypotheses of interest. The experimental effects in labeled MS studies include MS experiment and label. These effects should be chosen based on the study at hand, and may also include others such as LC column or laboratory technician in larger studies. Biological effects will be discussed further in a subsequent section.

The experimental effects are global terms, and are assumed to affect all proteins and peptides similarly. Thus, they should be estimated using all available data. However, due to the size of data sets generated from these experiments it is generally not possible with current computing infrastructure to fit the entire model at once. Thus, the model is broken into normalization and differential abundance pieces which are each fit separately. If good study design is utilized, then normalization and group effects are close to independent, allowing these to be estimated in two separate models to achieve the desired results. Due to the abundance-dependent data acquisition process, peptide must be included in the normalization model in order to estimate the normalization parameters properly [9,32]. Code to implement this via SAS is available from the authors. See the "Discussion" section for potential extensions to the normalization model.

#### Case study: GCM data

The GCM study had six MS experiments and four iTRAQ tags. Thus, experiment and tag are two known experimental effects to be included into the normalization model. Specimen is included as well to obtain a specimen-specific normalization. Thus, the normalization model on the additive scale is *log(y_ijkpm_) = expt_i _+ tag_j _+ spec_ij _+ pep_kpm _+ ε_ijkpm _*where model terms are as defined in the previous section. With the 2,637 unique peptides observed in this study, the matrix is too large to invert and as a result, even this normalization model must be fit iteratively as is generally the case with these studies. The normalized data are then the residuals from the normalization model, $y\text{\_}norm_{ijkpm}=log\left(y_{ijkpm}\right)-\left[e\hat{x}pt_{i}+tâg_{j}+spêc_{ij}\right]$ where the hat indicates estimated parameter values. The *pep_kpm _*term is not subtracted off since it is a biological effect and is included in the normalization only to appropriately line up the distributions between specimens. The normalization models for the other case studies contained the same terms.

**Figure 5:**
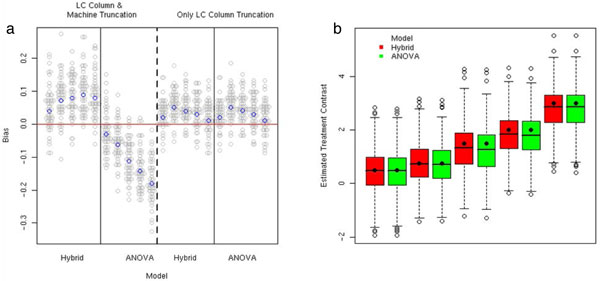
**Bias in parameter estimates**. Bias in MS experiment study group comparison estimates under two different mechanisms described in the text giving rise to missing data between MS experiments using either the ANOVA model normalization or a hybrid model incorporating censoring. A) Bias (vertical axis) is the difference between estimated and true MS experiment effects. The horizontal axis indexes varying MS experimental effects and analysis methods. B) Box and whisker plots of estimated study group differences. The dot indicates the true simulated difference.

We have investigated the utility of accounting for the abundance-dependent data acquisition, and therefore non-random missing data by incorporating a censoring mechanism into the normalization and differential abundance models [34]. iTRAQ-like data with either peptide competition alone or peptide competition plus a machine threshold for inducing missing data were simulated with MS experiment effects ranging from 0.5 to 2.0 and study group differences of 0.5, 1.0, 1.5, 2.0 and 2.5, all on the log2 scale. Incorporating a censoring mechanism into the modeling process reduces the bias in MS experiment effect estimation but does not reduce the variability in estimates (see Figure 5). However, due to the balance of study groups over MS experiments and tags in a properly designed study, the MS experiment effects cancel out in the class comparison calculation, resulting in essentially no difference in estimation of study group effects under the two models. Note that this does not imply that normalization is not necessary; it is still required to account for and therefore remove variability and improve reliability of treatment comparisons.

## Differential abundance

### Overview

Statistical models can be used to assess which peptides or proteins are significantly differentially abundant between study groups. The models are flexible, can accommodate nearly any experimental design, and consider the magnitude of signal relative to the variation in the data in order to determine whether the signal is appreciably larger than random noise. These methods have been shown to be the most powerful for hypothesis testing and enable estimates of fold change based on all available data. They are more straightforward than many ad hoc methods and result in simple summary statistics for each protein or peptide.

### Building the differential abundance model

We pick up the modeling discussion we began in the previous section where we discussed and demonstrated estimation and removal of the experimental effects. Now we turn our attention to the biological effects in the model. Differential abundance models are generally fit on a per-protein basis due to computational limitations. Thus, the differential abundance model reduces to *y_norm_ijkpm _= grp_k _+ pep_kpm _+ ε_ijkpm_*. The hypothesis test of *grp_k _*is of greatest interest, as this is a measure of the difference in abundance between the two groups relative to the noise in the data. Research has shown use of all peptide information associated with a protein without summarization in a statistical model is more efficient than ad hoc summaries or decision rules [35].

### Variance structure

It is important to understand the variance structure or precision in your data as this has implications for the statistical models and estimation strategies used. We and others have found that precision is generally a function of mean abundance in iTRAQ data [11,36-40]. This varying precision is not evident in standard residual plots, but is evident in per-MS experiment plots. The variance structure will likely depend on the MS technology used. Thus, this should be examined for each study to determine the structure and appropriate modeling approaches in light of this (See the "Estimation of model parameters and calculating significance" section).

#### Case study: yeast data

**Figure 6:**
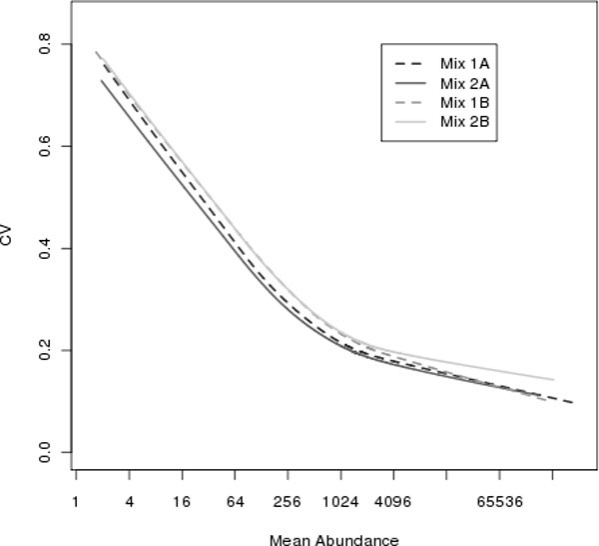
**CV as a function of protein abundance**. Within experiment peptide coefficient of variation (CV) on the vertical axis versus average abundance on the horizontal axis for the yeast data. The line is a moving average smoother indicating average CV as a function of mean abundance.

We demonstrate the mean-variance relationship graphically. The within MS experiment coefficient of variation (CV), which corresponds to the standard deviation on the raw scale, plotted versus the mean abundance demonstrates that precision increases as abundance increases (see Figure 6). We have observed this relationship in several iTRAQ data sets produced from human and yeast specimens on Orbitrap and TOF mass spectrometers. It is important to look at your data to understand the correct modeling procedure to use.

### Estimation of model parameters and calculating significance

When variance or precision is constant, ordinary least squares (OLS) are used to estimate model parameters. However, as shown in the previous section, precision can be abundance-dependent in iTRAQ data. Thus, other means must be used for parameter estimation. Including MS scan, i.e., elution time, in the model to account for varying precision results in a saturated model. Thus, weighted least squares (WLS) is used to estimate model parameters. In WLS, each abundance value is given a weight that is inversely proportional to the precision. As a result, peptides measured with more precision are given more weight in the analysis, whereas those measured with less precision are given less weight. The weight can be estimated theoretically using the relationship between the Gaussian and Lognormal distributions. Alternatively, it can be estimated empirically. We have chosen to use an empirical estimate, assigning each peptide the value of the moving average smoother at its abundance value on a CV plot such as that in Figure 6. In these data, this weighting accounts for the variability due to differences in elution time.

It is not computationally feasible to estimate all parameters within the biological model simultaneously. Thus, in practice, differential abundance models are fit on a per-protein or per-peptide basis depending on the goals of the study at hand. We focus on per-protein level models here. In biological terms, fitting models on a per-protein basis allows estimation of the amount of random variability for each protein separately rather than forcing it to be the same across all proteins.

**Figure 7:**
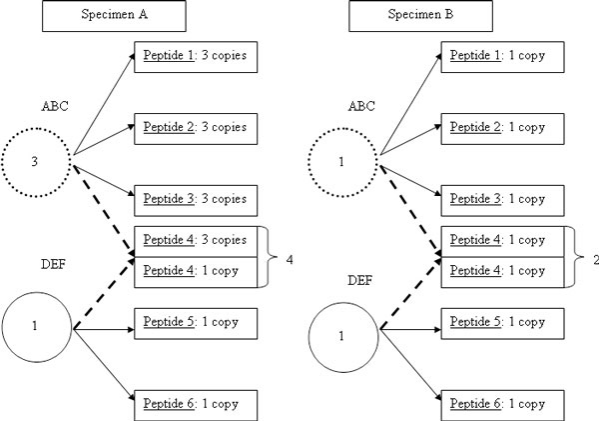
**Cartoon illustration of the impact of including peptides mapped to multiple proteins in relative quantification**. As described in the text, the inclusion of including peptides which are mapped to multiple proteins results in biased estimates of fold changes. Therefore, these peptides are generally included in the normalization step, but excluded from the relative quantification step.

Peptides mapped to multiple proteins are not included in differential abundance models. Shared peptides, peptides that are present in more than one protein, are common in shotgun proteomic experiments. These shared peptides have been found to be beneficial to determine the presence of a protein [41]. However, these same shared peptides can become problematic in estimating relative abundance of a protein. A simple example is demonstrated in Figure 7 containing two specimens, each of which contain two proteins which are represented by solid or dotted line circles. The true relative ratios for Specimen A to Specimen B are 3:1 and 1:1 for proteins ABC and DEF, respectively, and peptide 4 is shared between both proteins. If the shared peptide is ignored, the fold change difference between Sample A and B for protein ABC is simply $\frac{3+3+3}{1+1+1}=\frac{9}{3}=3$ and for DEF is $\frac{1+1}{1+1}=\frac{2}{2}=1$ which match the true fold changes. However, after the identification process Peptide 4 will be assigned a total abundance of 4 in Specimen A and 2 in Specimen B, and these abundance values will be attributed to both proteins in the resulting output. The resulting fold change estimates for ABC and DEF now become $\frac{3+3+3+4}{1+1+1+2}=\frac{14}{5}=2.8$ and $\frac{1+1+4}{1+1+2}=\frac{6}{4}=1.5$, respectively. Thus, both estimates of fold change for the proteins are biased away from their true values as a result of including the shared peptide. For this reason, when doing quantitative analyses, peptides that appear in more than one protein are excluded from analysis.

Due to the large number of proteins being examined in global mass spectrometry studies, stringent criteria must be used to determine significance of a peptide. One strategy is to use the Bonferroni correction which involves computing a significance threshold based on the number of proteins being tested as 0.05/(the number of proteins being tested). This is generally accepted to be too stringent and frequently results in no significant proteins. The distribution of p-values can be used to compute an expected false discovery rate (FDR) [42,43]. These numbers, called q-values, give an indication of the level of significance in the study. An FDR value is the number of genes among those declared to be significant which are expected to be falsely declared significant. A study resulting in a uniform distribution of p-values (which would be expected by chance under the null hypothesis of no differences between the study groups) will have large FDR values. However, a study with a skewed distribution of p-values having a spike near zero will have smaller FDR values.

### Visualizing and interpreting significance and fold changes

Digesting the volumes of data resulting from a high dimensional study can be challenging. Here we present some visualization and computational tools we have found helpful for drawing biological conclusions.

#### Case study: GCM data

Recall the primary goal of the GCM study was to compare abundance for proteins between four types of subjects, GCM, DM, LM and normal controls. We focus on the GCM versus DM comparison as an example. Note that due to the fact that the normal controls were pooled prior to mass analysis, it is not possible to properly estimate biological variability within this group. The differential abundance model was fit in SAS [44] with the following commands:

proc mixed data=abundance;

by protein_id;

class dx_grp;

model logYnorm=dx_grp;

/*This performs all pair wise comparisons between diagnostic groups*/

lsmeans dx_grp/pdiff;

ods output diffs=dx_grp_contrasts;

ods output tests3=overallFtest;

run;

A few lines of the output listing are shown in Table 4. The "Accession" column is the protein name. The "Comparison" column indicates which groups are being compared and which group is in the numerator for the fold change estimate. The "Estimate" column is the model estimate of the difference between GCM and DM on the log2 scale. The "Standard error" column contains the standard error of this estimate, and is an indicator of the precision associated with the comparison. The "Fold Change" column is 2 raised to the power in the "Estimate" column, so 2^-2.068 ^in the first row of the table. 95% confidence interval limits for the fold change are the next two columns and the p-value is contained in the last column.

**Table 4:** Differential abundance output.

Accession	Comparison	Estimate	Standard Error	Fold Change	Lower 95^th ^CI	Upper 95^th ^CI	P-value
hCP1788782	GCM/DCM	-2.068	0.1272	0.238	0.186	0.306	2.09E-27
hCP1887960	GCM/DCM	1.894	0.08586	3.717	3.142	4.399	2.65E-18
hCP1780445	GCM/DCM	1.145	0.08317	2.211	1.878	2.602	9.99E-17
1OPH_A	GCM/DCM	-2.764	0.2218	0.147	0.095	0.227	1.27E-16
AAH78670.1	GCM/DCM	2.156	0.1805	4.458	3.130	6.350	1.51E-15
AAF29581.1	GCM/DCM	-3.013	0.266	0.124	0.074	0.207	5.60E-13

This table shows sample differential abundance output from the top 5 proteins when ranked by p-value in the GCM study. Columns are explained in the text.

**Figure 8:**
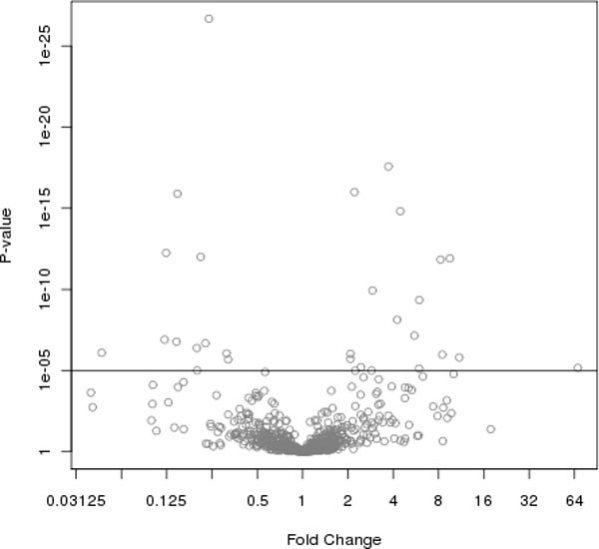
**Volcano plot**. A volcano plot from the GCM study demonstrating magnitude and significance of the protein comparisons between the GCM and DM groups. The vertical axis indicates -log10(p-value). The horizontal axis indicates log2 fold change, here labeled on the fold change scale.

A volcano plot helps to understand the level of significance and magnitude of changes observed in the study as a whole (see Figure 8). The fold change on the log2 scale is placed on the horizontal axis (sometimes labeled on the log2 scale, sometimes labeled on the fold-change scale) and p-value on the -log10 scale is placed on the vertical axis. Points on the plot tend to look like lava spewing from a volcano, hence the name. Points nearest the far right and left hand sides of the plot have the largest fold changes while those along the top of the plot are the most statistically significant. Thus, these may help one to use both fold change and significance in determining which proteins to carry forward for further study based on both statistical and biological criteria.

**Figure 9:**
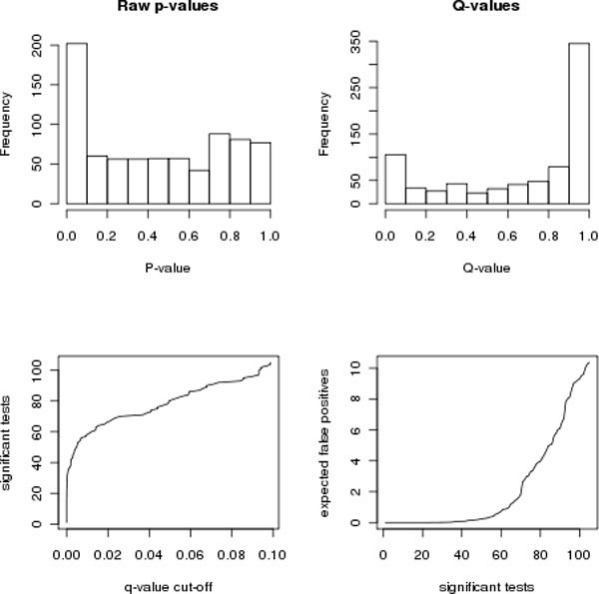
**Visualization of statistical significance in the GCM study**. A) Histogram of the p-values. B) Histogram of the q-values (FDR values). C) Number of tests declared to be significant (vertical axis) as a function of the FDR cut-off used (horizontal axis). D) Expected number of false positive tests (vertical axis) as a function of the number of significant tests (horizontal axis).

While plots of p-values and FDR rates cannot help to distinguish true and false positive test results, they are useful for understanding the likelihood of real change. If there are no differences between the two groups, a uniform distribution of p-values would be expected. The presence of the spike for small p-values indicates that there are more significant differences than would be expected by chance (see Figure 9a). An FDR value (or q-value) for a given protein, indicates the expected number of false positive tests if the p-value for that protein is used as the significance cut-off (see Figure 9b). Figures 9c and 9d can help determine an acceptable significance threshold in light of the number of expected false discoveries. In this particular example, a q-value threshold of 2% would result in approximately 60 expected false positive tests (see Figure 9c). On the other hand, if approximately the top 70 proteins are declared significant, one of these is expected to be a false positive (see Figure 9d).

**Figure 10:**
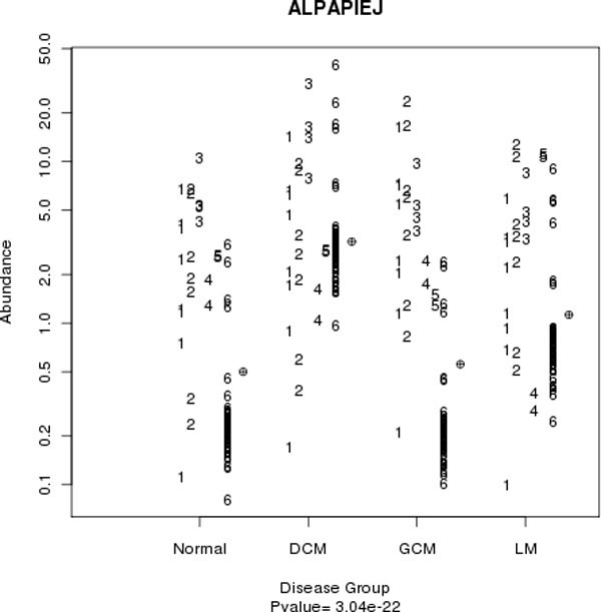
**Dot plot for peptide with sequence ALPAPIEJ in the GCM study**. The vertical axis indicates abundance on the log2 scale. The horizontal axis indicates study group. Numbers in the plot indicate the MS experiment in which the peptide was detected. The circles with + inside to the right of the points for a given study group indicate the mean for that study group. While this peptide has a small p-value, it appears that observations in run 6 are driving the significance. Relying on p-value alone isn't enough; one needs to look at data for a complete interpretation.

Summary statistics such as estimates of fold change and p-values are useful. However, it is wise to also look at the data being summarized. A dot plot is useful for visualizing the behavior of the peptides within a given protein, and understanding the underling variability (see Figure 10). At least one study group is statistically significantly different from the other groups in this example peptide dot plot, but there is still a lot of variability in the underlying peptide distributions. There is substantial overlap in the abundance distribution between study groups, indicating this peptide may not be a good biomarker of disease. This particular peptide was detected in all six MS experiments; this is not the case for all peptides.

## Discussion

In this work, the primary focus has been on the iTRAQ labeling protocol, but the basic statistical principles highlighted here are directly applicable to other experiments which utilize different labeling protocols. What does vary between labeling protocols is the mathematical model governing the labeling process which ultimately dictates the analytical methods used to quantify relative abundance information from the raw data. Thus, each labeling protocol will require different analytical methods. For example, in the case of ^16^O/^18^O stable isotope labeling, all peptides mixed in heavy water would be shifted two Daltons to the right of those mixed in light water (^18^O has two extra neutrons, thus is 2 Daltons heavier) and peak picking algorithms would be used to identify these provided that 100% of the oxygen atoms were fully exchanged. However, due to less than pure ^18^O water, naturally occurring isotopes, and a probabilistic model governing the oxygen exchange rates, some of the labeled mixture will have 0, 1 or 2 extra neutrons. Regression modeling strategies can be used to tease apart just how much came from the light and heavy samples, respectively [45,46]. Coupled with sound statistical practices, a full understanding of the labeling protocol being used and the necessary analytical steps to follow will maximize the information content of the experiment.

There is evidence that the variance is a function of mean abundance as discussed in the "Differential abundance" section. The analytical strategy demonstrated herein utilized that information in the differential abundance models by using WLS as the estimation technique. However, the normalization models were estimated via OLS which does not account for the varying levels of precision. Ideally both of these models would incorporate the weighting. This poses computational challenges since the entire model, normalization plus differential abundance, cannot be fit at once with current computing resources. Incorporation of the weighting into both steps would require iterating between estimation of normalization parameters and differential abundance parameters and is work that requires further investigation.

The models described herein are considered "fixed" effect models. It may be desired to utilize a "mixed" effect model in which some effects are considered fixed while others are considered to be random. Likely random effects are subject and peptide. Designating subject as a random effect would broaden the scope of inference from only the subjects selected for the current study to the population of subjects the sample represents. Designating peptide as a random effect acknowledges that due to the data-dependent acquisition process, the same peptides may not be observed every time. Use of global experimental factors as random effects in the normalization model is currently problematic due to computational limitations and the fact that iterative estimation processes are not yet worked out for random effects. Fixed effect models have been shown to have greater sensitivity than mixed effect models, and therefore more desirable in discovery studies whereas properties of the mixed effect models make them more attractive for studies validating results [35].

## Conclusions

Use of replication, randomization and blocking in the process of experimental design for labeled MS studies can avoid confounding of experimental and biological effects and minimize variability. A statistical model can be used to account for experimental and biological sources of variation to describe the observed data and produce unified estimates of changes between study groups along with associated measures of uncertainty.

## Abbreviations

MS: mass spectrometry; SILAC: stable isotope labeling by amino acids in cell culture; iTRAQ: isobaric tag for relative and absolute quantitation; SCX: strong cation exchange; GCM: giant cell myocarditis; DCM: idiopathic dilated cardiomyopathy; LM: lymphocytic myocarditis; LC: liquid chromatography; ADT: androgen deprivation therapy; LTQ: linear trap quadrupole; RBD: Randomized Block Design; MVA: Minus versus Average; CV: coefficient of variation; OLS: ordinary least squares; WLS: weighted least squares; FDR: false discovery rate.

## Competing interests

The authors declare that they have no competing interests.
